# Structure of Nora virus at 2.7 Å resolution and implications for receptor binding, capsid stability and taxonomy

**DOI:** 10.1038/s41598-020-76613-1

**Published:** 2020-11-12

**Authors:** Pasi Laurinmäki, Shabih Shakeel, Jens-Ola Ekström, Pezhman Mohammadi, Dan Hultmark, Sarah J. Butcher

**Affiliations:** 1 HiLIFE-Institute of Biotechnology, University of Helsinki, Viikinkaari 9, P.O. Box 56, 00014 Helsinki, Finland; 2 Molecular and Integrative Biosciences Research Programme, Faculty of Biological and Environmental Sciences, University of Helsinki, Viikinkaari 9, P.O. Box 56, 00014 Helsinki, Finland; 3 Department of Molecular Biology, Umeå University, 901 87 Umeå, Sweden; 4 Institute of Biosciences and Medical Technology, BioMediTech, University of Tampere, 33014 Tampere, Finland; 5 Present Address: MRC Laboratory of Molecular Biology, Francis Crick Avenue, Cambridge, CB2 0QH UK; 6 Present Address: VTT Technical Research Centre of Finland Ltd., 02044 Espoo, Finland

**Keywords:** Viral proteins, Virus structures, Evolution, Genetics, Microbiology, Molecular biology, Structural biology

## Abstract

Nora virus, a virus of *Drosophila*, encapsidates one of the largest single-stranded RNA virus genomes known. Its taxonomic affinity is uncertain as it has a picornavirus-like cassette of enzymes for virus replication, but the capsid structure was at the time for genome publication unknown. By solving the structure of the virus, and through sequence comparison, we clear up this taxonomic ambiguity in the invertebrate RNA virosphere. Despite the lack of detectable similarity in the amino acid sequences, the 2.7 Å resolution cryoEM map showed Nora virus to have T = 1 symmetry with the characteristic capsid protein β-barrels found in all the viruses in the *Picornavirales* order. Strikingly, α-helical bundles formed from the extended C-termini of capsid protein VP4B and VP4C protrude from the capsid surface. They are similar to signalling molecule folds and implicated in virus entry. Unlike other viruses of *Picornavirales*, no intra-pentamer stabilizing annulus was seen, instead the intra-pentamer stability comes from the interaction of VP4C and VP4B N-termini. Finally, intertwining of the N-termini of two-fold symmetry-related VP4A capsid proteins and RNA, provides inter-pentamer stability. Based on its distinct structural elements and the genetic distance to other picorna-like viruses we propose that Nora virus, and a small group of related viruses, should have its own family within the order *Picornavirales*.

## Introduction

Nora virus is a positive-sense, single-stranded RNA virus infecting different *Drosophila* species including *Drosophila melanogaster* and *Drosophila simulans*^1^. The virus infects the midgut cells and the infection is transmitted vertically by the fecal–oral route. Interestingly, the infected insects exhibit no obvious pathological effects^2–5^. Nora virus has several characteristic features of the order *Picornavirales*^6^, such as an isometric capsid about 30 nm in diameter and a replicative cassette that includes an RNA helicase, a protease and an RNA-dependent RNA polymerase^1^. Based on the conserved polymerase and helicase sequences, Nora virus is similar to viruses in the *Picornavirales* families *Iflaviridae*, *Secoviridae*, *Picornaviridae* and *Dicistroviridae*^1,7,8^. However, uniquely, the Nora virus genome is organised into four open reading frames (ORF), and for a *Picornavirales* virus it has a relatively large RNA genome of 12,333 nucleotides^1,9^ (Fig. S1). It shares these characteristics with a small group of Nora-like viruses, most of them isolated from different insects^1,7,8,10,11^.

Nora virus ORF4, situated at the 3′-terminal end of the genome, encodes a 931 amino acid-long polyprotein. The translated polyprotein is cleaved at two positions (264 aa and 515 aa) post-translationally by a virus-encoded protease to form the virion’s mature structural proteins VP4A (29 kDa), VP4B (28 kDa) and VP4C (48 kDa)^5,9^. The proteins encoded by ORF4 have no significant sequence similarity to the capsid proteins of other picorna-like viruses or to each other. However, structure predictions indicated a possible picorna-like jelly roll fold for VP4A and VP4B. In contrast, VP4C was predicted to have a mainly α-helical secondary structure^1^. Furthermore, VP4C is susceptible to proteolysis and is fragmented in virions isolated from fly feces, which begs the question whether it has a structural role or not^9^. Additionally, trace amounts of the VP3 protein from ORF3 have been identified in purified Nora virus particles, isolated from feces. VP3 is not required for virus assembly, but stabilizes the capsid against heat and protease treatment. It is unclear whether or not it is physically integrated into the capsid^9,12^. We present the Nora virus capsid structure at 2.7 Å resolution, using electron cryo-microscopy (cryoEM) and icosahedral image reconstruction, which helps us to understand the architecture of this virus, to investigate the presence of VP3 in the capsid and to investigate the function of VP4C.

## Results and discussion

**Figure 1:**
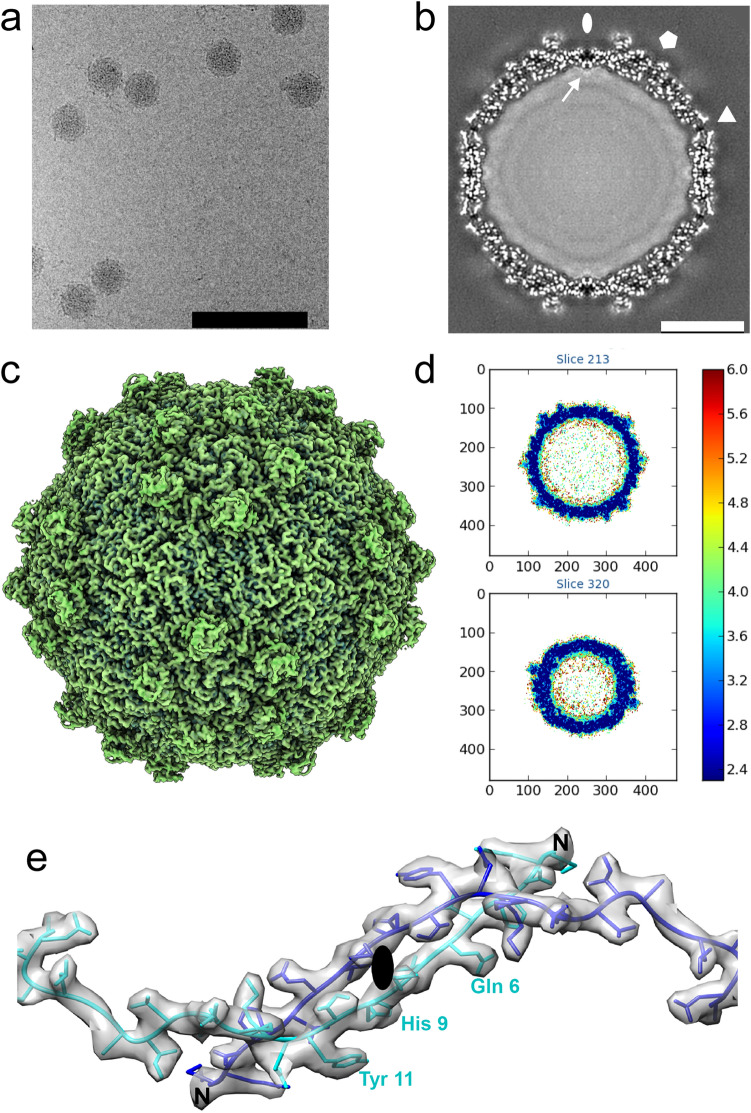
Nora virus cryoEM reconstruction. (**a**) Representative cryoEM micrograph of Nora virus. Scale bar is 100 nm. (**b**) Central section through the Nora virus reconstruction with protein white. White ellipse, pentagon and triangle indicate positions of symmetry axes. White arrow: strongest ordered RNA density under the twofold symmetry axis. Scale bar is 10 nm. (**c**) Isosurface representation of Nora virus density rendered at 3 SD above the mean. (**d**) Local resolution of the EM density map calculated using ResMap^36^, 2 slices each of 1.06 Å thick are shown. The upper slice is 28.62 Å from the center of the particle, the lower slice is 84.8 Å from the center of the particle (which would be section 240) Color key units are in Å. (**e**) Representative atomic model fit of VP4A in the vicinity of the two-fold symmetry axis. Grey semi-transparent surface: reconstructed volume rendered at 3 standard deviations above the mean. Cyan and blue ribbons: atomic models of 25 N-terminal residues of two symmetry related VP4As. N-termini marked with a letter N, three AA residues are annotated for one VP4A.

**Figure 2:**
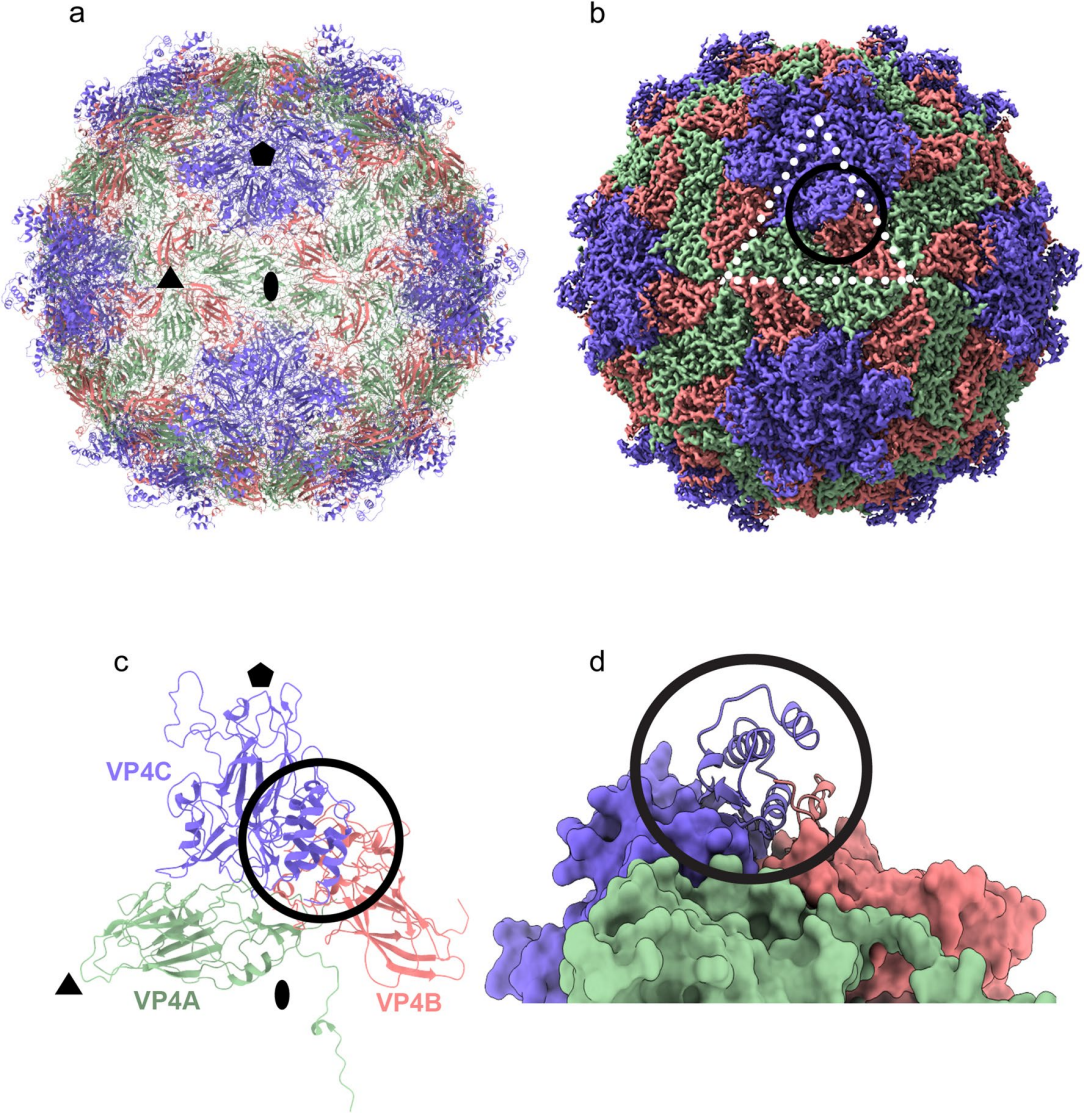
Architecture of Nora virus capsid. (**a**) Atomic model of Nora virus capsid in ribbon. (**b**) Isosurface representation of Nora virus density rendered at 3 SD above the mean. The map was zoned and coloured according to model with distance of 5 Å using chimeraX. (**c**) Capsid asymmetric unit in ribbon. (**d**) Close-up of surface protrusion shown as ribbon. Green: VP4A; pink: VP4B; and blue: VP4C. Black ellipse, pentagon and triangles indicate positions of two-fold, five-fold and three-fold symmetry axes in (**a**) and (**c**). The surface protrusion is encircled in (**b**)–(**d**).

We determined a 2.7 Å resolution Nora virus structure using electron cryo-microscopy and single particle image analysis (Fig. 1). The models of capsid proteins VP4A, VP4B and VP4C were built de novo to generate an atomic model of Nora virus constrained by the density from the reconstruction (Fig. 2 and Table 1). The Nora virus reconstruction displays an icosahedrally- symmetric particle with a *T* = *1* (pseudo *T* = *3*) triangulation number; a capsid architecture previously described as one of the main characteristics of the order *Picornavirales* (Fig. 1 c and Fig. 2a)^6^. The Nora virus capsid asymmetric unit is built from three subunits, a single copy each of VP4A, VP4B and VP4C (Fig. 2c). Major domains of all the three subunits are β-sheet jelly rolls (Fig. 2a,c)^6^. The five-fold vertices are composed of VP4C, the three-fold facets are composed of VP4A and VP4B and the two-fold edges are composed of VP4A (Fig. 2a,b). We were unambiguously able to trace the C-α backbone for VP4A (residues 1–249 out of 264), for VP4B (2–242 out of 251) and for VP4C (1–364 out of 416). The disordered C-termini regions are presumably exposed on the capsid surface, as the last visible residues contribute to the striking protrusions that circle the pentamers composed of both the VP4B and the VP4C C-termini. Hence, the termini are potentially susceptible to host protease attack, explaining the observed shortening of VP4C in virus isolates from fly feces^9^. No additional density for VP3 was identified in the capsid density. As only trace amounts of VP3 have been detected previously in isolated virus, if associated with the capsid, it is probably in non-stoichiometric amounts and may also be disordered, resulting in it being averaged out during icosahedral averaging.

**Table 1:** CryoEM data collection and model refinement statistics.

	Noravirus			
**Data collection and processing**				
Voltage (keV)	300			
Electron exposure (e^−^/Å^2^)	40			
Defocus range (µm)	0.5–1.5			
Pixel size	1.06			
Symmetry imposed	I			
Number of movies collected	3516			
Final particle images (no.)	16,131			
Map resolution (Å)	2.7			
Map resolution range (Å)	2.4–6			

	VP4A	VP4B	VP4C	VP4ABC
**Refinement**				
Map cross correlation (around atoms)	0.855	0.813	0.789	0.805
rmsd (bonds)	0.01	0.01	0.01	0.01
rmsd (angles)	1.08	1.33	1.18	1.27
All-atom clashscore	2.87	3.16	3.24	3.49
Ramachandran outliers	0.81%	0.84%	1.10%	0.83%
Ramachandran allowed	7.29%	13.81%	11.88%	12.15%
Ramachandran favoured	91.90%	85.36%	87.02%	87.03%

### The evolutionary expansion of the Nora virus capsid

**Figure 3:**
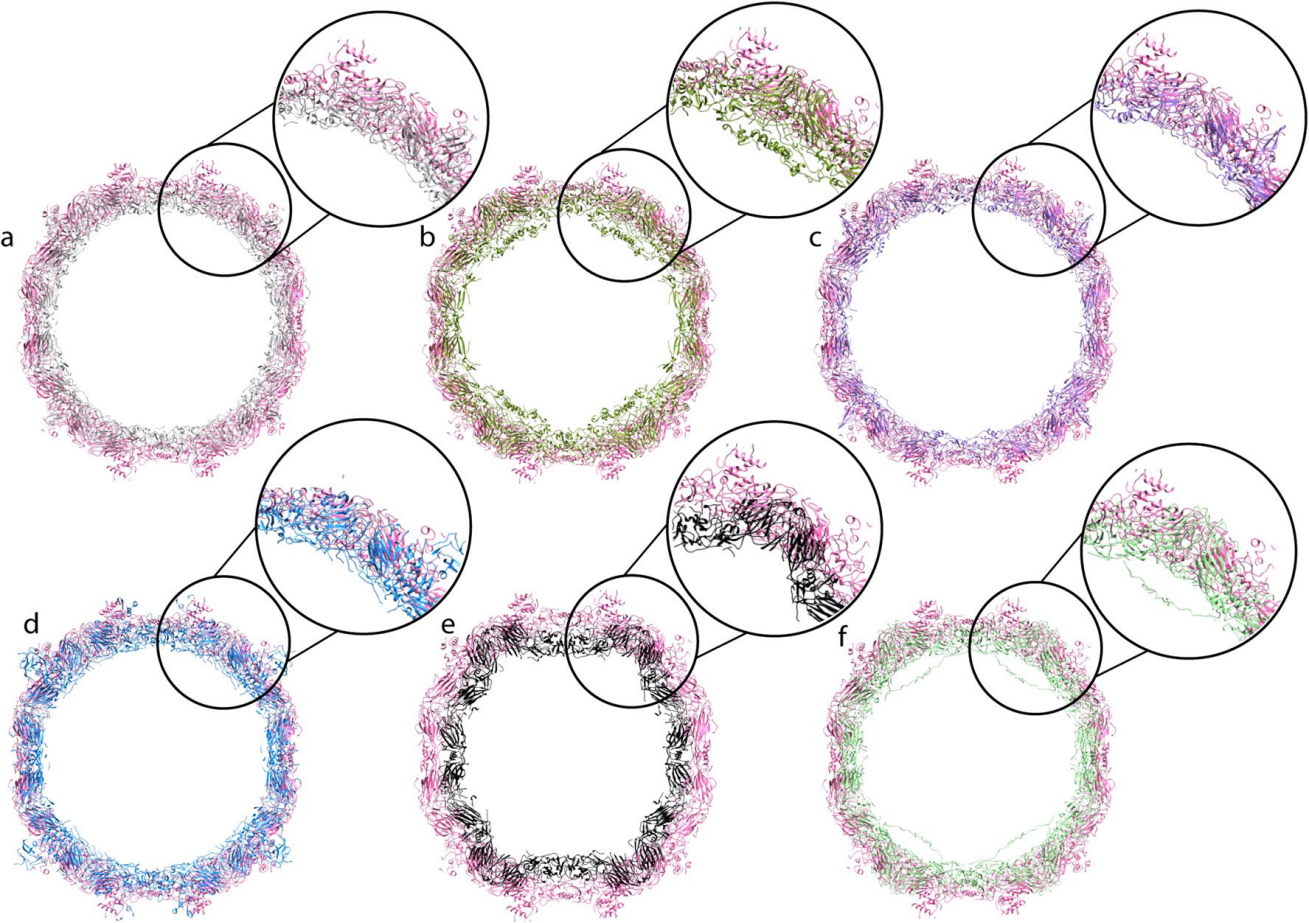
Capsid size comparison. Central cross-sections of Nora virus capsid (pink) versus (**a**) Triatoma (PDB: 3nap; *Dicistroviridae*; grey), (**b**) cricket paralysis virus (PDB: 1b35; *Dicistroviridae*, dark green), (**c**) Israel acute paralysis virus (PDB: 5cdc; *Dicistroviridae*, purple), (**d**) slow bee paralysis virus (PDB: 5j98; *Iflaviridae*, blue), (**e**) cowpea mosaic virus (PDB: 1ny7; *Secoviridae*, black), (**f**) coxsackievirus A9 (PDB: 1d4m; *Picornaviridae*, green). Nora virus has a larger diameter, thinner, capsid shell than the other viruses which is most pronounced around the two-fold axis, e.g. on the equator in this view. The β-barrels of VP4C around the vertices lie flatter on the capsid surface compared to equivalent barrels of picornaviruses. Overall, the capsid comparison shows that the Nora virus has undergone drastic expansion by means of translation as well as rotation of capsid proteins.

The Nora virus capsid size was compared with the capsids from representative viruses within the order *Picornavirales*: *Dicistroviridae, Iflaviridae, Secoviridae and Picornaviridae*. The inner shell of the Nora virus capsid had the highest diameter of all of them (Table 2, Fig. 3), in line with the need to accommodate the unusually large 12 kb genome. The volume available inside the capsid per nucleotide of the genome varied from 0.52 to 0.67 (Table 2). Interestingly, the buried surface area of the Nora virus capsid is smaller than all the others, suggesting a relative expansion of the capsid during evolution accomplished through conformational change (Table 2, Fig. 3). Additionally, the VP4C β-barrels around the vertices lay flatter on the capsid surface compared to the more tangential arrangement of β-barrels in the other viruses (Fig. 3). This translation of the β-barrels appears to be the most obvious cause of capsid expansion in Nora virus. In conclusion, the capsid size comparison shows that while there is significant variation in the capsid sizes of *Picornavirales*, the Nora virus capsid is the largest thus far. The expansion allows the encapsidation of the large Nora virus genome. There are potentially many other factors that could also affect the packaging density, as the percentage difference in the ratio of genome length to capsid volume is not linear. One such possibility is that the secondary and tertiary structure of the RNA varies. Another possibility is that the presence of counterions may affect the density. A third possibility is that the number of amino acid residues actually included in the atomic models may not account for the full-length protein present in the capsids, thus the volume allocated to the genome maybe overestimated. In the case of Nora virus only one missing amino acid residue in VP4B is thought to be internal, the other missing amino acids in all three proteins are thought to be external. Hence, the volume occupied by 60 amino acids in total would reduce the volume available to the Nora virus genome. If trace amounts of VP3 are present within the capsid, these too would reduce the volume available to the genome.

**Table 2:** Comparison of capsid size inner volumes, genome sizes and buried surface area among representative viruses of *Picornavirales*.

Family^a^	Genus	Virus name	PDB id	Inner diameter (nm)	Shell thickness (nm)	Inner volume (10^3^ nm)	Genome size^b^ (kb)	Volume per nucleotide (nm^3^)	Buried surface area (10^3^ nm)
Unclassified	Unclassified	Nora virus	5mm2	24.2	10.1	7.4	12.5	0.59	2.0
*Dicistroviridae*	*Triatovirus*	Triatoma virus	3nap	22.8	10.8	6.2	9.2	0.67	2.5
*Dicistroviridae*	*Aparavirus*	Israel acute paralysis virus	5cdc	22.8	11.6	6.2	9.7	0.64	2.3
*Dicistroviridae*	*Cripavirus*	Cricket paralysis virus	1b35	21.6	12.8	5.3	9.4	0.56	3.0
*Iflaviridae*	*Iflavirus*	Slow bee paralysis virus	5j98	22.8	14.6	6.2	9.5	0.65	2.1
*Picornaviridae*	*Enterovirus*	Coxsackievirus A9	1d4m	19.6	13.6	3.9	7.6	0.52	2.6

^a^Cowpea mosaic virus is not included in this table as it contains a bipartite genome with each genome packed in a separate capsid. ^b^200 nucleotides are added to each genome for the poly-A tail.

### Potential receptor binding region

**Figure 4:**
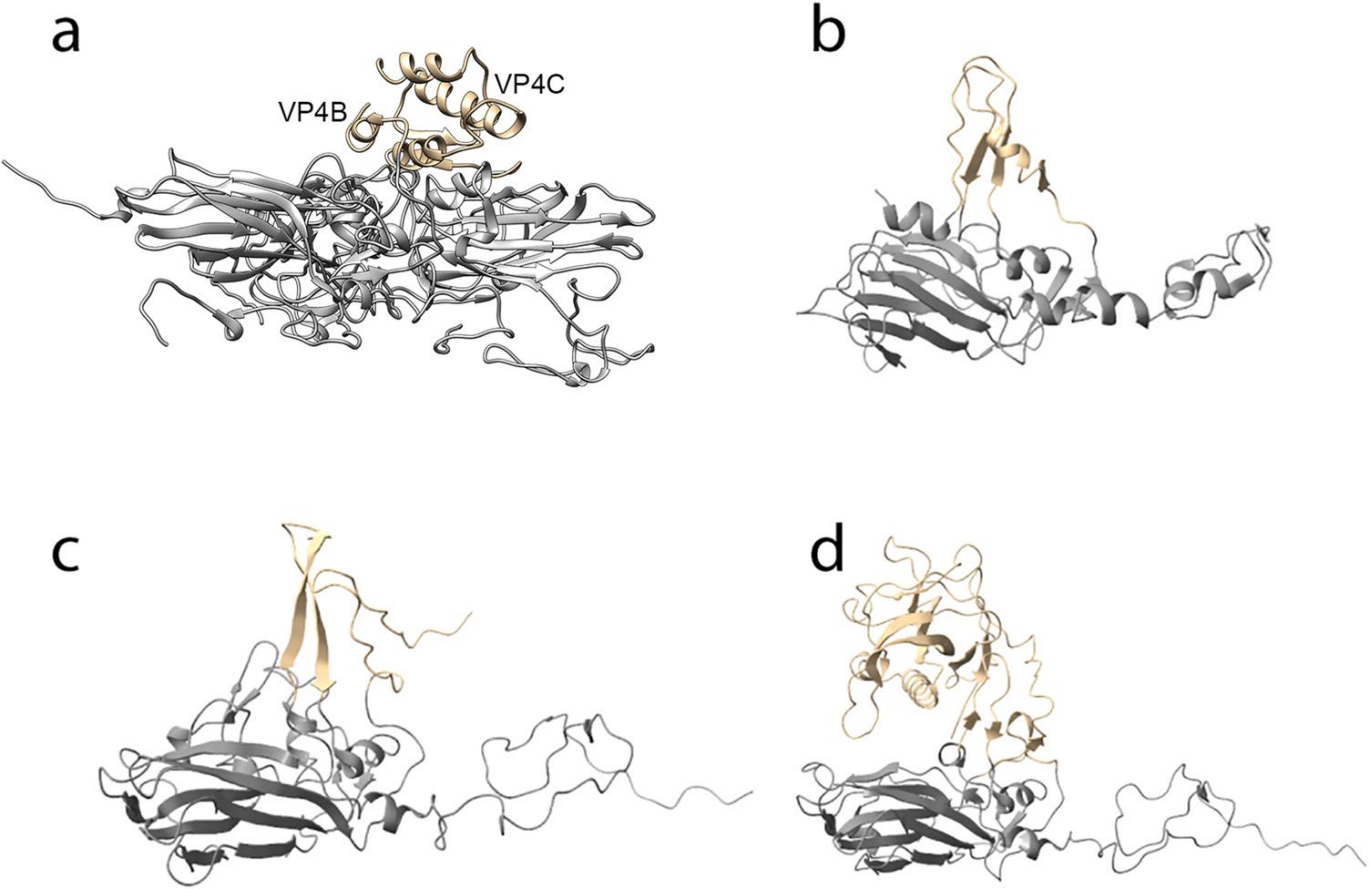
Surface protrusion comparison. The surface protrusions (gold) on the (**a**) Nora virus, (**b**) triatoma virus, (**c**) Israel acute paralysis virus and (**d**) slow bee paralysis virus are significantly different from each other. The Nora virus VP4C and VP4B C-termini form an α-helix-bundle. Triatoma has the least pronounced protrusion, made by VP1, a capsid protein present around the vertices. It is composed of β-hairpin and a short helix. Israel acute paralysis virus has long β-strands protruding out from the capsid protein VP2 found around the three-fold axes. The capsid protein VP3 C-terminus in slow bee paralysis virus forms a surface protrusion made of β-sheets and two helices. The VP3 is protein present around the three-fold symmetry axes. Only the position in Nora virus and triatoma virus is similar.

Structural alignment with DALI (13) showed that VP4A is most similar to FMDV VP2 (Z-score: 12.8; rmsd: 3.0; PDB: 1fmd; family: *Picornaviridae*; genus: *Aphthovirus*), VP4B is most similar to human Aichi virus VP0 (Z-score: 10.6; rmsd: 3.2; PDB: 5gka; family: *Picornaviridae*; genus: *Kobuvirus*) and VP4C is most similar to hepatitis A virus VP3 (Z-score: 11.0; rmsd: 3.2; PDB: 4qpg; family: *Picornaviridae*; genus: *Hepatovirus*). The β-barrels account for the alignment. However, the most prominent capsid surface features are the 60 mainly α-helical protrusions that are situated at the interface between VP4C contributing most of the residues and VP4B contributing one α-helix (Fig. 2b–d). These surface protrusions are significantly different from that of other insect viruses: triatoma virus, Israel acute paralysis virus and slow bee paralysis virus where the surface protrusions mostly consist of β strands (Fig. 4). We entered the structured VP4C residues 287–364 into a DALI alignment. Strikingly, it had structural similarity to inositol 1, 4, 5-triphosphate receptor type 1 (PDB: 3UJO), α-*N*-acetylglucosaminidase (PDB: 4XWH) and the non-structural ORF 12 of the virulent lactococcal phage p2 (PDB: 3D8L). All these three molecules are involved in signalling, suggesting that the Nora virus surface protrusion may have a similar role, binding to a cell surface receptor and causing downstream signalling to bolster virus entry. Additionally, the surface charge distribution of these protrusions shows a positive patch which, similar to the C-terminal extension of S protein in cowpea mosaic virus, may have a role in capsid assembly by stabilizing the formation of pentamers during assembly^14^.

### Stabilization of the capsid

**Figure 5:**
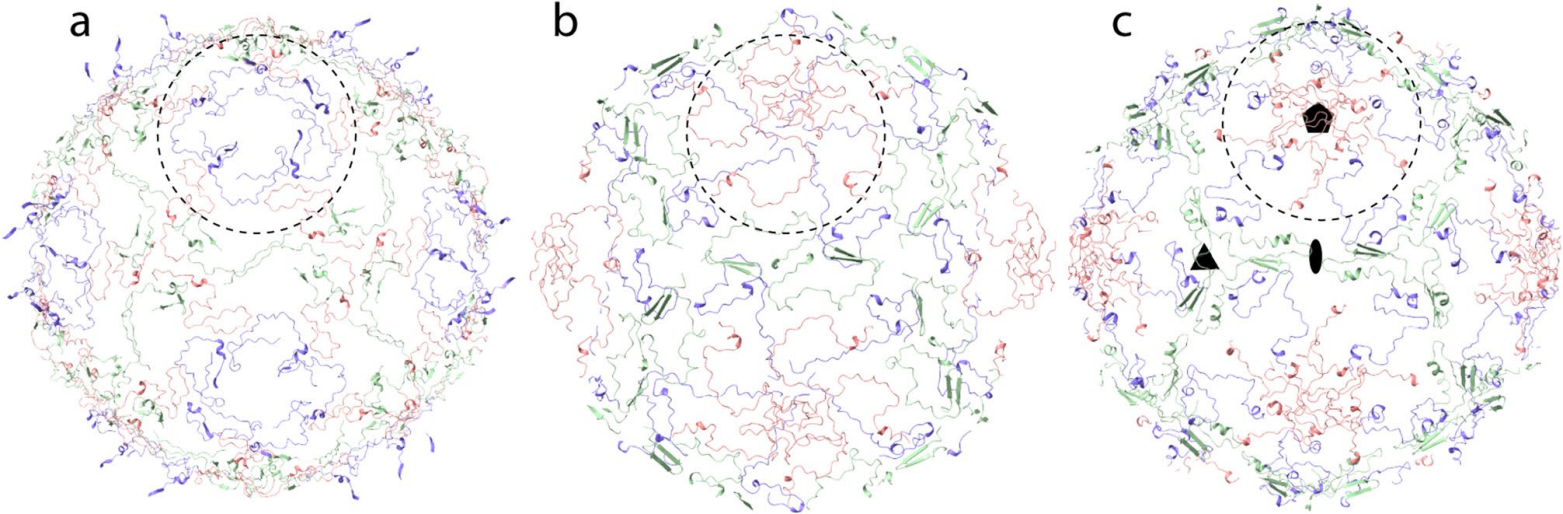
Network of capsid protein N-termini. The first 50 N-terminus residues present in the atomic models of Nora virus (**a**), CVA9 PDB ID: 1d4m (**b**) and HPeV1 PDB ID 4z92 (**c**) are shown. Green ribbon is VP4A in Nora virus, VP1 in CVA9 and VP0 in HPeV1. Pink ribbon: is VP4B in Nora virus and VP3 in CVA9 and HPeV1. Blue ribbon is VP4C in Nora virus and VP1 in CVA9 and HPeV1. Only the front half of the capsids are shown for clarity. Black ellipse, pentagon and triangles indicate positions of two-fold, five-fold and three-fold symmetry axes in (**a**)–(**c**). The dotted circle indicates the region which stabilizes the pentamer within the capsid.

A very distinct feature in Nora virus is the lack of the annulus found below the vertices in dicistrovirus, picornavirus and iflavirus capsids formed by interaction of 5 N-termini from the VP3 capsid proteins. Annulus formation is a primary requirement for intra-pentamer stability in those capsids. It is fulfilled in Nora virus by extensive interaction of VP4B and VP4C N-termini around the five-fold but at a much greater distance from the five-fold axis of symmetry (Fig. 5).

**Figure 6:**
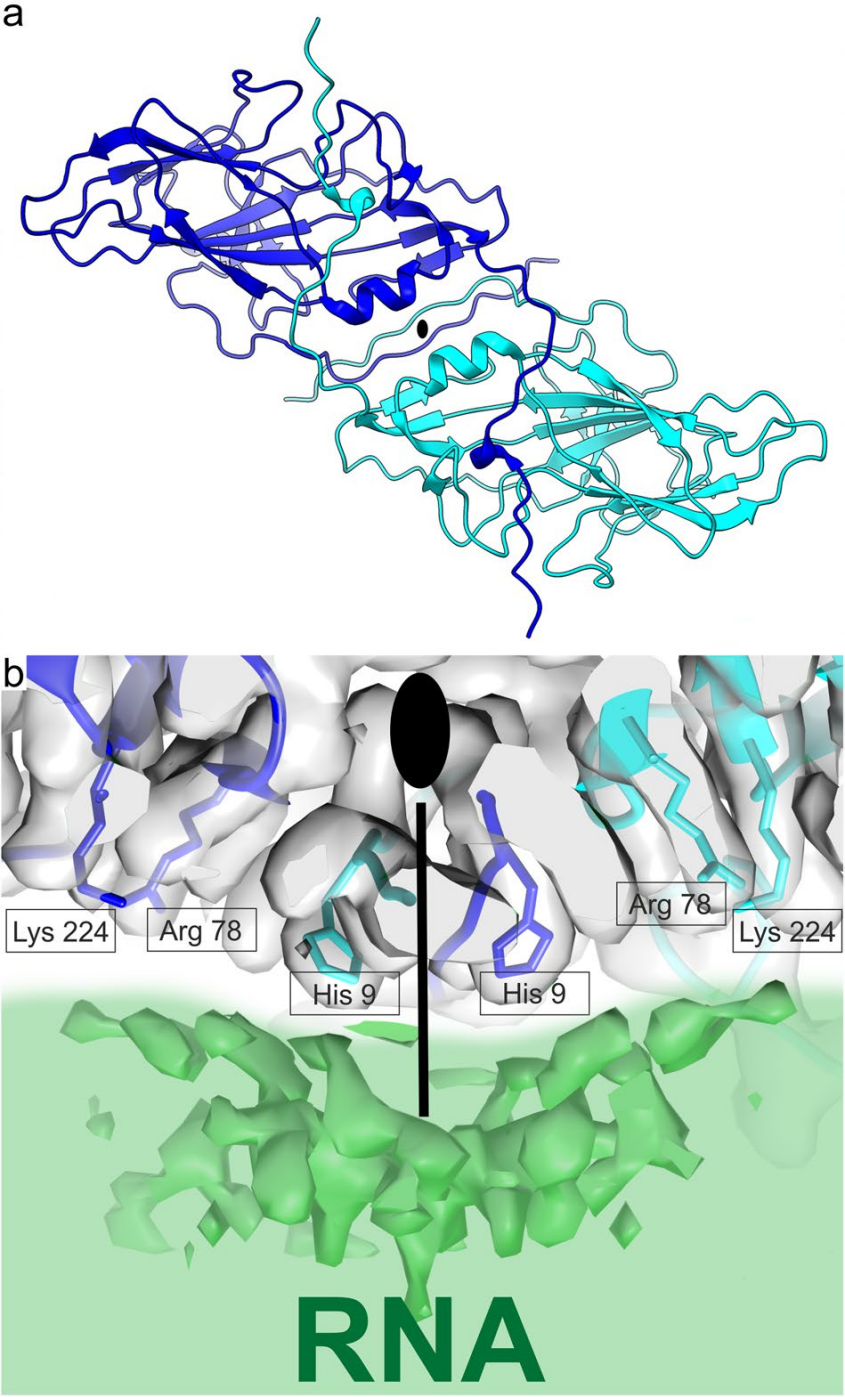
Two-fold symmetry axis. (**a**) VP4A dimer shown from outside the capsid. (**b**) VP4A interaction with RNA; a close-up view of the area depicted by white arrow in Fig. 1b showing interaction between VP4A and RNA genome below the twofold symmetry axis. Grey semi-transparent surface: reconstructed VP4A volume rendered at 2.5 SD above mean. Green surface: RNA-density rendered at 2.5 SD above mean. The green wash represents the interior of the capsid. (**a**,**b**) Cyan and blue ribbon: atomic models of the two twofold associated VP4A-chains, positively charged VP4A side chains are shown and labelled. Black ellipse and line indicate position of the twofold symmetry axis.

The Nora virus capsid seems to utilize the N-termini of VP4A to provide interpentamer stability by spanning from the three-fold axis to the two-fold axis, a feature lacking in most other viruses of *Picornavirales* except human parechoviruses (HPeVs)^13,14^. However, unlike HPeVs, there is a crossover of the VP4A N-terminus (residues 1–13) with a symmetry-equivalent N-terminus of another VP4A from the neighbouring pentamer at the two-fold axis (Fig. 1e). Additionally, two molecules of VP4A sit either side of the two-fold axis of symmetry. At this position, there are two α-helices (aa 109–117), one contributed by each molecule on the capsid surface (Fig. 2c). Although this α-helical feature is common in other *Picornavirales*, in Nora virus, they are further apart (Fig. 6a), for instance, the middle residue in the helix, T114 CB is 6.9 Å from its symmetry related atom in comparison to 4.6 Å for Q94 CB of VP2 in coxsackievirus A 9 (CVA9). These helices are important for capsid stability and separate during RNA egress in *Picornaviridae*^15–18^. In Nora virus, the interface between the VP4A molecules is strengthened by the tight interaction of the VP4A N-termini in the inner surface (Figs. 1e, 6a). Such deviation from the common theme raises the question of how the genome release occurs in Nora virus, and this will require further investigation.

### Capsid protein–RNA interactions

The symmetry-related, positively-charged histidines (residue number 9) of the two VP4A N-termini on the two-fold axis closely interact with the ssRNA genome. This causes a localised condensation of RNA density in this region around the twofold symmetry axis (Figs. 1b, 6). Arginine (residue number 78) and lysine (residue number 224) add additional positive charge in close proximity, thereby further stabilizing the electrostatic interactions between the capsid protein and the RNA (Fig. 6). This location of RNA condensation is in contrast to the one found in HPeVs where it occurs around the vertices^19,20^. RNA–protein contacts around the twofold symmetry axes have been reported in several *Picornavirales* including cowpea mosaic virus containing RNA-2, in rhinovirus A2 and in CVA9. However, not with this particular distribution^16,21,22^. Hence, both the viral assembly and RNA release process may occur by different means compared to other *Picornavirales*. In the future, the RNA structure could possibly be studied further by asymmetric reconstruction of the capsids to high resolution, but the current data set was too small for this endeavour, probably requiring at least tenfold more particles as was done for bacteriophage MS2^23^.

### Phylogenetic analysis

**Figure 7:**
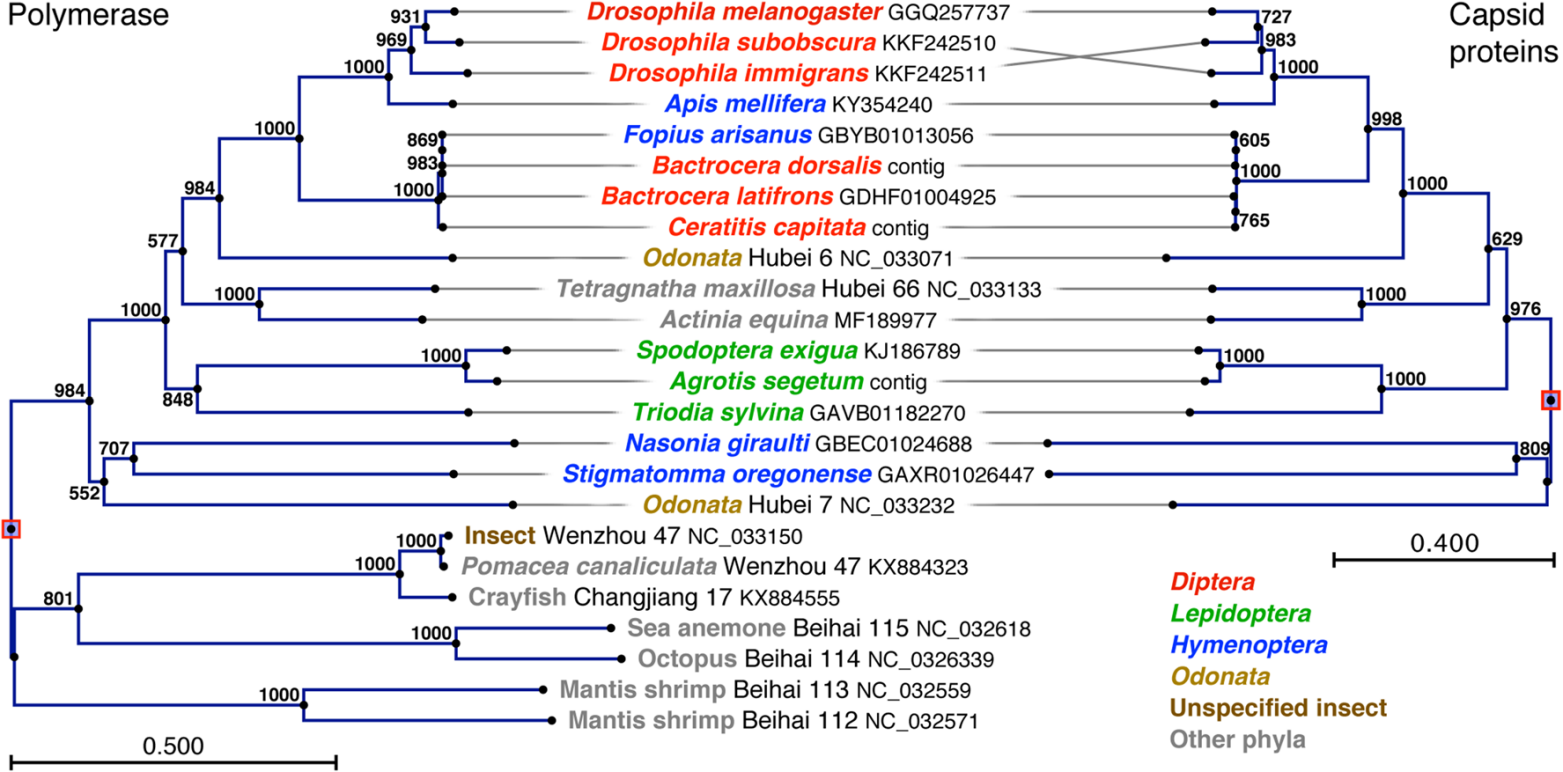
Phylogenetic analysis of Nora-like viruses. Nora VP4-like sequences were retrieved from the NCBI nr/nt, EST and TSA databases by pblast and tblastn searches. If possible, contigs were constructed from overlapping short TSA and EST sequences. Single short partial sequences were excluded from further analysis. Phylogenetic trees were reconstructed from conserved regions in the polymerase (left) and VP4 (right) amino acid sequences, using the neighbor-joining algorithm of the CLC Main Workbench package, version 6.7.1. As an outgroup for the polymerases we used members of the Nora Virus Related Clade, described by Shi et al.^8^, that lack Nora VP4-like capsid proteins.

Sequences related to the Nora virus capsid proteins can be identified in a number of virus-like sequences in the databases (Fig. 7 and Fig. S1). Based on the conserved RNA-dependent RNA polymerase sequences, Shi et al.^8^ have previously defined a “Nora Virus Related Clade” of viruses, but we found that Nora-like capsid protein sequences are only present in a subset of these viruses. This Nora-like subset forms a well-defined monophyletic group with a conserved genome organization (Fig. 7 and Fig. S1a), which is different from other members of the clade (Fig. S1b). The Nora-like viruses have generally been isolated from insects, with the exception of one isolate from a spider (*T. maxillosa*) and one from a sea anemone (*A. equina*). The capsid protein sequences are well conserved among the members of this group, suggesting that the structure described here is well adapted to the genome size and/or the biology of these viruses.

The phylogenetic tree of the Nora-like viruses tends to mirror that of their hosts (Fig. 7), suggesting that this virus family is old and that the viruses tend to keep narrow host ranges. Occasional shifts in host range must have happened, the most dramatic one involving a sea anemone. It should be stressed, though, that most of these sequences come from large metagenomic projects, and the exact links between the viruses and the organisms in which they were found are still uncertain. For instance, *Fopius arisanus* is a parasitoid wasp, feeding on *Bactrocera dorsalis* and *Ceratitis capitata*, and the virus may well derive from the gut contents of this wasp. Similarly, dragon flies (*Odonata*) are voracious predators on flies and other flying insects, and their viromes may include viruses present in their diet. A similar argument could be made about the spider. It is of course also possible that the viruses have adapted to replicate in these predators. However, the sea anemone remains a mystery.

In conclusion, we showed that the Nora virus has a T = 1 arrangement where VP4C is present around the five-fold axes of symmetry, VP4A around the two-fold axes of symmetry and VP4B around the three-fold axes of symmetry. Each protein shows the β-jelly roll fold characteristic of *Picornavirales*, but with an α-helical domain protrusion from the virion surface and an unusual interaction of two N-termini from symmetry-related VP4A around the two-fold axes. Taken together, both global and detailed analysis of the capsid structure, the genome organization and the genetic distance to other viruses, suggest that Nora virus and the clade of related viruses can be described as representatives of a new virus family within the order *Picornavirales*.

## Materials and methods

### Production and purification of Nora virus

Persistent viral infections are common in *Drosophila*^24^. To avoid cross-contamination with other viruses, dechorionated *Drosophila melanogaster* eggs were infected with Nora virus as described earlier^9^. Nora virus was propagated and purified essentially as described earlier^1–3,9^.

### CryoEM and image processing

Aliquots (3 μl) of purified virus in 10 mM Tris–HCl pH 7.4. buffer were vitrified in a Leica EM GP device at 22 °C and 70% humidity on glow discharged Quantifoil 2/2 holey carbon grid in liquid ethane. CryoEM data were collection at eBIC at the Diamond Light Source, UK on a FEI Krios 300 kV TEM equipped with a Gatan post-GIF K2 Summit detector. The GIF was set to 20 eV slit width and FEI EPU software was used to automatically the data. Each exposure was written out as a 20 frames stack with an estimated total electron dose of 40 e^−^/Å^2^ and a sampling of 1.06 Å/pixel. The initial dataset consisted of 3516 frame stacks, and the frames were aligned prior to processing using motioncorr^25^. We used Ethan^26^ for automated particle picking, CTFFIND4 for CTF estimation and correction^27^. An initial model was built with 150 picked particles using random model generation module in AUTO3DEM^28^ with icosahedral-symmetry imposed. Both 2D and 3D classification in Relion version 1.3^29^, were used to reduce the dataset heterogeneity. A total of 16,131 particles were refined using the 3D autorefine module, followed by particle polishing, an additional 3D autorefine step, and finally B-factor correction in the “post processing” module using a B-factor value of − 20. The final refinement step combining two independent datasets gave a resolution of 2.7 Å as assessed by the 0.143 criterion Fourier shell correlation from the deposited half maps using the EMDB server (EMDB-3528; Fig. S2).

### Model building and refinement

We used the “Volume tracer”-tool in UCSF Chimera^30^ to trace C-α backbones of the constituent proteins from capsid density. Volumes corresponding to the three distinct subunit densities were then segmented with “Zone”-tool in UCSF Chimera at a radius of 8 Å. The models were built de novo into each segmented EM density in COOT^31^ using the known amino acid sequences of VP4A, VP4B and VP4C. The models were refined in real space using Phenix^32^ and in Fourier space using Refmac^33^. This step was iterated with local refinements in COOT until no further improvement in the refinement statistics were observed (Table 1). We combined the atomic models of the three subunits into the full asymmetric unit, which was consequently re-refined in Phenix to resolve any clashes within the asymmetric unit. The UCSF Chimera sym command was used to build the whole virus capsid. UCSF Chimera or ChimeraX^34^ were used for all visualizations.

### Structural alignments and buried surface area calculation

The structural alignments were performed on the DALI web server^13^. The buried surface area for all the viruses were obtained from VIPERdb association energy data analysis^35^.

## Supplementary information

Supplementary Figures.

## Data Availability

The datasets generated during the current study are freely available in the following public repositories: the density map of the Nora virus reconstruction has been deposited in the Electron Microscopy Data Bank under the accession codes EMD-3528 https://www.ebi.ac.uk/pdbe/entry/emdb/EMD-3528. The atomic models for Nora virus have been deposited in the Protein Databank in Europe with the PDB ID: 5mm2 https://www.ebi.ac.uk/pdbe/entry/pdb/5mm2. All the raw data collected for the Nora virus reconstruction are available through the Electron Microscopy Pilot Image Archive under the accession code EMPIAR-10088 https://www.ebi.ac.uk/pdbe/emdb/empiar/.
