# Supplementary material for: Structure of Nora virus at 2.7 Å resolution and implications for receptor binding, capsid stability and taxonomy
Source: Sci Rep. 2020 Nov 12;10:19675. doi: 10.1038/s41598-020-76613-1 (PMC7661533; doi:10.1038/s41598-020-76613-1)
Supplement: Supplementary file 1 — Supplementary Figures. [file 41598_2020_76613_MOESM1_ESM.pdf]

Supplementary Information

Structure of Nora virus at 2.7 Å resolution and implications for receptor binding, capsid stability and taxonomy.

Pasi Laurinmäki[a,b], Shabih Shakeel[a,b], Jens-Ola Ekström[c,d], Pezhman Mohammadi[a], Dan Hultmark[c,d], Sarah J. Butcher[a,b*]

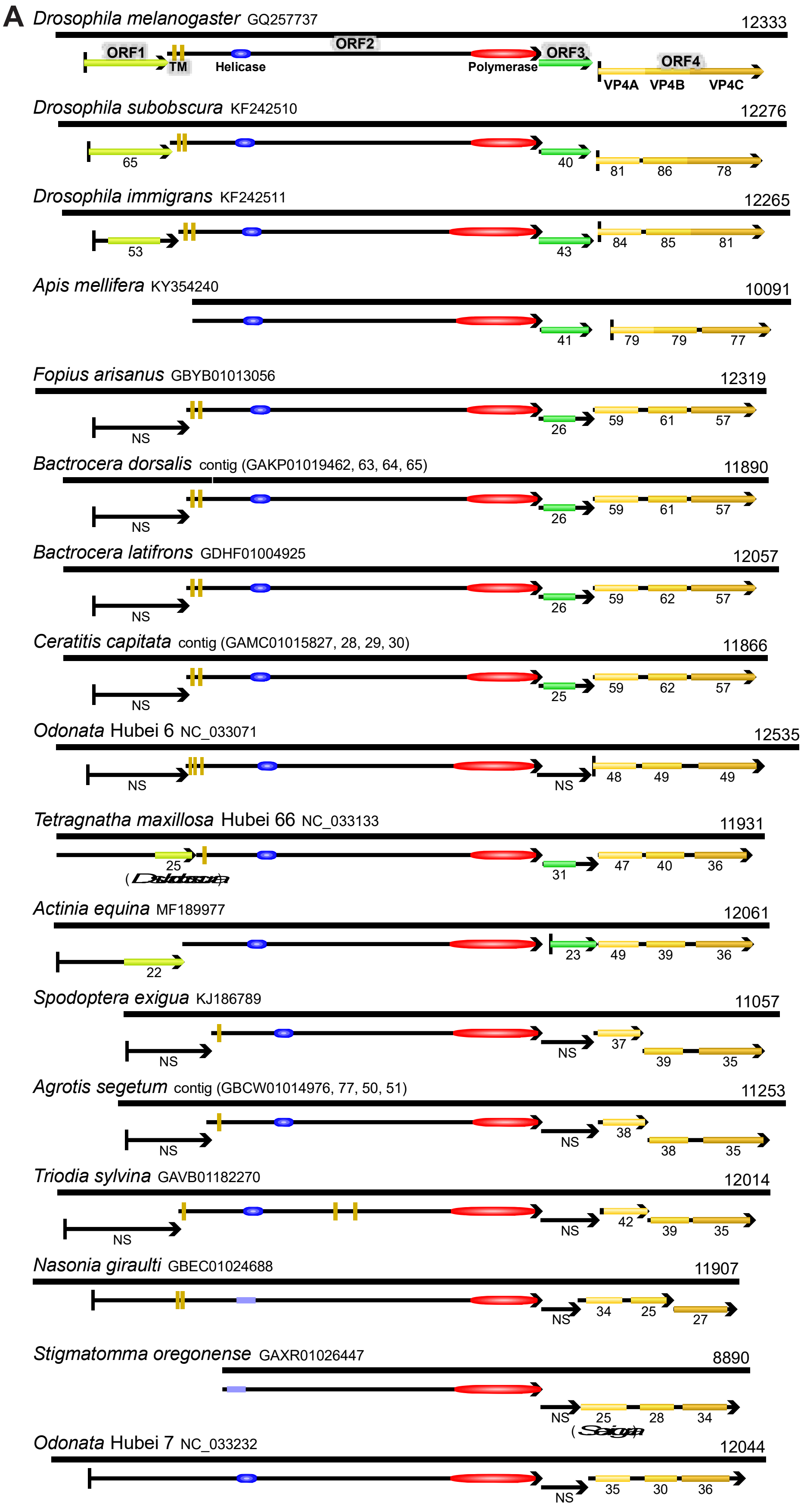
A
Drosophila melanogaster GQ257737
12333
ORF1
TM
Helicase
ORF2
Polymerase
ORF3
ORF4
VP4A VP4B VP4C
Drosophila subobscura KF242510
12276
65
40
81 86 78
Drosophila immigrans KF242511
12265
53
43
84 85 81
Apis mellifera KY354240
10091
41
79 79 77
Fopius arisanus GBYB01013056
12319
NS
26
59 61 57
Bactrocera dorsalis contig (GAKP01019462, 63, 64, 65)
11890
NS
26
59 61 57
Bactrocera latifrons GDHF01004925
12057
NS
26
59 62 57
Ceratitis capitata contig (GAMC01015827, 28, 29, 30)
11866
NS
25
59 62 57
Odonata Hubei 6 NC_033071
12535
NS
NS
48 49 49
Tetragnatha maxillosa Hubei 66 NC_033133
11931
25
31
47 40 36
Actinia equina MF189977
12061
22
23
49 39 36
Spodoptera exigua KJ186789
11057
NS
NS
37
39 35
Agrotis segetum contig (GBCW01014976, 77, 50, 51)
11253
NS
NS
38
38 35
Triodia sylvina GAVB01182270
12014
NS
NS
42
39 35
Nasonia giraulti GBEC01024688
11907
NS
34 25
27
Stigmatomma oregonense GAXR01026447
8890
NS
25 28 34
Odonata Hubei 7 NC_033232
12044
NS
35 30 36

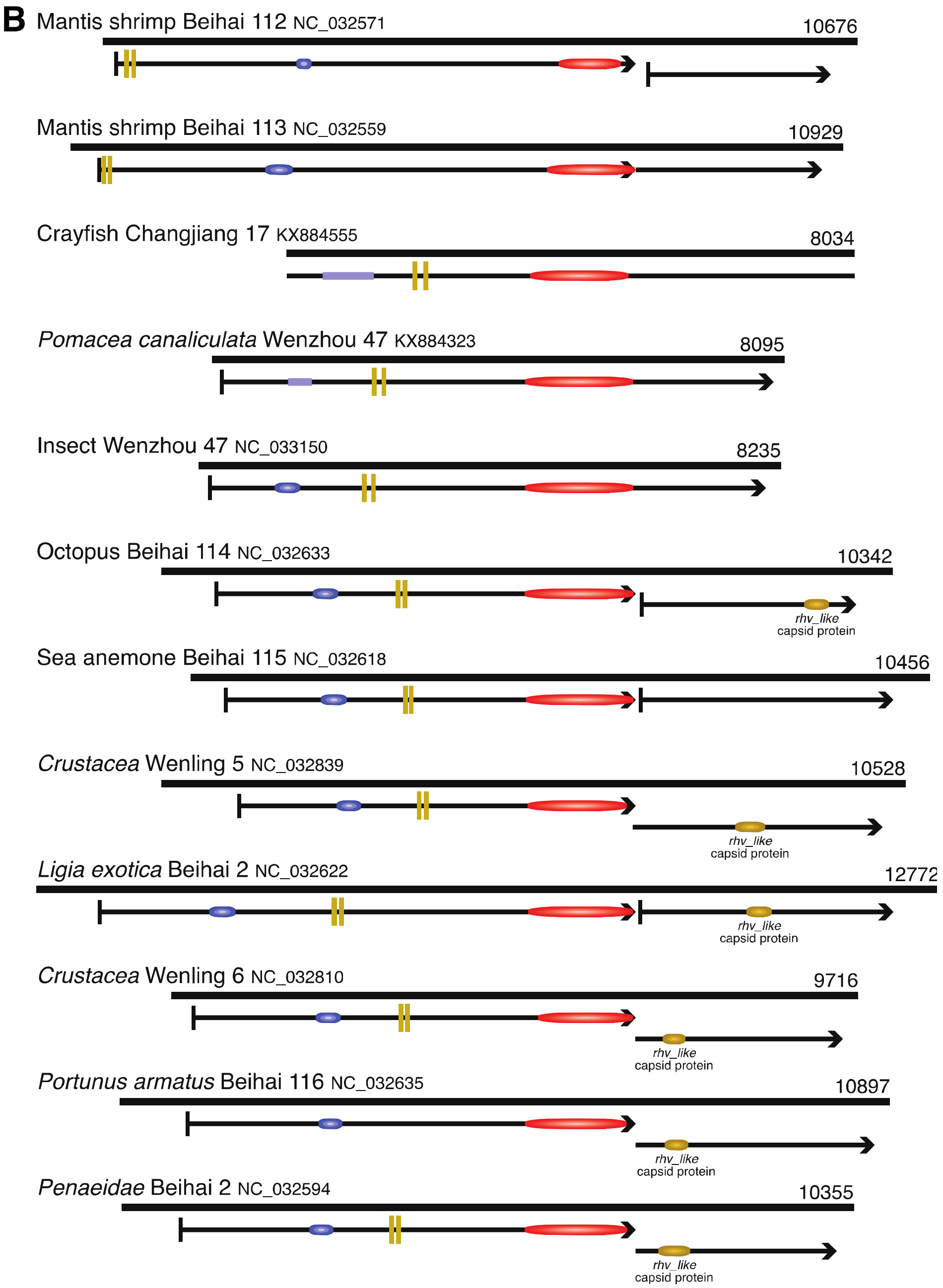

Figure S1. Genome organization of Nora-like viruses and their relatives. A. Open reading frames in the Nora-like viruses described in Fig. 7 are shown as arrows. Regions with amino acid sequence similarity (E<1) to the *D. melanogaster* Nora virus in ORFs 1, 3 and 4 are shown as colored bars, and the percent amino acid sequence identity is indicated below. In ORF2, only the conserved CDD helicase (RNA_helicase, pfam00910, **blue**) and polymerase (RdRP_1, pfam00680, **red**) domains and predicted transmembrane regions (**TM**) are indicated. **B.** Genome organization of other members of the Nora Virus Related Clade described by Shi *et al.* (8). No regions with amino acid sequence similarity (E<1) to the *D. melanogaster* Nora virus ORF1, 3 or 4 were found in these viruses, but *rhv_like* picornavirus capsid protein domains (cd00205) were identified in some of them.

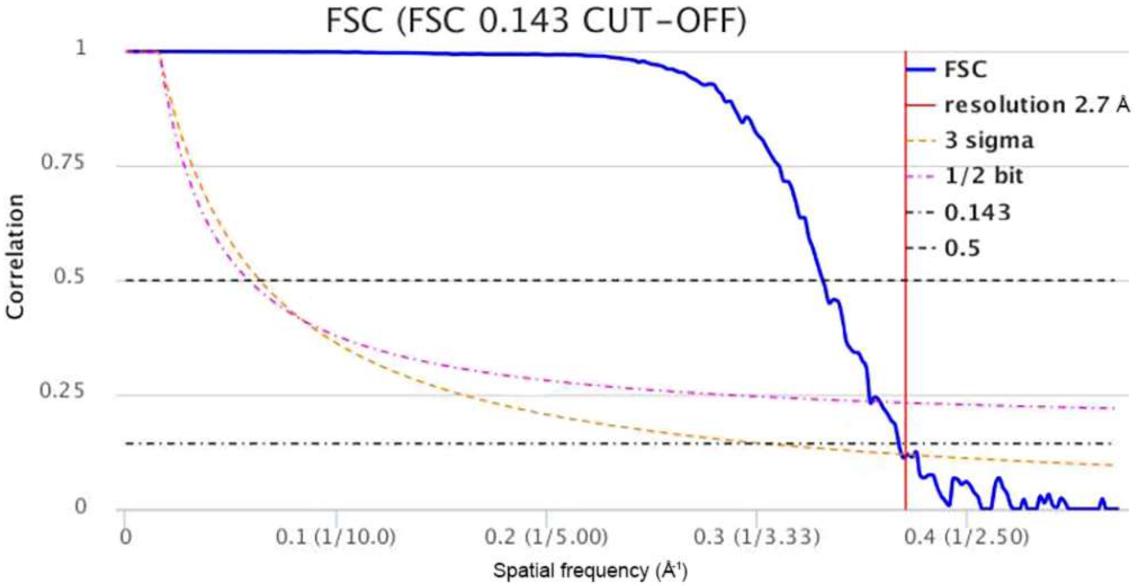

Figure S2. Fourier Shell Correlation (1; extracted from the Electron Microscopy Data Bank accession code EMD-3528 http://www.ebi.ac.uk/pdbe/entry/emdb/EMD-3528)

References

1. G. Harauz, M. van Heel Exact filters for general geometry three dimensional reconstruction (1986) Optik 73:146-156
